# Supplementary material for: Genetic Variants Determine Treatment Response in Autoimmune Hepatitis
Source: J Pers Med. 2023 Mar 17;13(3):540. doi: 10.3390/jpm13030540 (PMC10052918; doi:10.3390/jpm13030540)
Supplement: Supplementary file 1 [file jpm-13-00540-s001.zip › jpm-2249265-SI.pdf]

Supplementary information

Supplementary Table S1. The distribution of variant alleles in comparison to an ideal population in the Hardy–Weinberg equilibrium. Levels of significance: * p<0.05 (Chi-square test).

	**PNPLA3**	**MBOAT7**	**TM6SF2**	**HSD17B13**
**Homozygous reference (n)**	40	25	67	41
**Heterozygous (n)**	31	38	14	29
**Homozygous variant (n)**	10	18	0	11
**Variant allele frequency**	0.31	0.46	0.09	0.31
**Chi-squared value**	1.93	0.24	0.76	3.02
**Chi-squared test *p*-value**	0.16	0.62	0.38	0.08

Abbreviations: PNPLA3, patatin-like phospholipase domain-containing protein 3; MBOAT7, membrane-bound O-acyltransferase domain containing 7; TM6SF2, transmembrane 6 superfamily member 2; and HSD17B13, 17-beta-hydroxysteroid dehydrogenase 13.

Supplementary Table S2. Characteristics of patients stratified according to PNPLA3-rs738409 status, wildtype (C/C) vs. heterozygous (C/G) vs. homozygous (G/G) risk allele. Levels of significance: * p<0.05 (Mann–Whitney U test, Chi-square test).

	C/C (n=40)	C/G (n=31)	G/G (n=10)	*p*-value
**Sex (m/f)**	6/34 (15.0% m)	13/18 (41.9% m)	4/6 (40.0% m)	0.03*
**Age (years)**	55.3 (47.2-62.0)	58.0 (48.8-70.2)	45.6 (24.9-59.1)	0.072
**Cirrhosis at BL (n)**	4 (10.0%)	4 (12.9%)	3 (30%)	0.25
**BMI (kg/m2)**	23.4 (21.3-27.0)	25.7 (23.4-27.6)	25.4 (22.0-26.0)	0.247
**Cholesterol (mg/dl)**	191.5 (162-232)	176.1 (142.9-246.0)	163.4 (139.3-189.9)	0.259
**CRP (mg/dl)**	0.4 (0.1-0.8)	0.7 (0.1-1.3)	0.5 (0.3-1.0)	0.336
**Hemoglobin (g/dl)**	13.7 (12.4-14.4)	14.2 (13.4-14.8)	13.2 (12.6-14.4)	0.377
**WBC (G/L)**	5.2 (4.4-6.3)	6.6 (5.0-8.2)	6.3 (5.0-7.1)	0.053
**Thrombocytes (G/L)**	201 (176-242)	202 (159-240)	195.5 (128.5-238.8)	0.830
**Albumin (x LLN)**	1.2 (1.1-1.3)	1.2 (1.0-1.3)	1.2 (1.1-1.2)	0.680
**Prothrombin time (%)**	90.7 (74.8-98.7)	79 (68.4-89)	95.5 (77.4-107.6)	0.149
**ALT (x ULN)**	9.8 (2.8-19.0)	14.9 (3.2-34.4)	8.8 (2.9-14.2)	0.738
**Bilirubin (x ULN)**	1.3 (0.7-2.9)	1.8 (0.7-6.9)	0.7 (0.5-1.8)	0.104
**APRI after 1 year**	0.4 (0.3-0.6)	0.4 (0.3-0.6)	0.5 (0.4-0.9)	0.202
**IgG (mg/dl)**	1905 (1407-2650)	1905 (1427.5-2587.5)	2610 (1465-3447.5)	0.661
**Diabetes mellitus (n)**	4/40 (10.0%)	0/31	1/10 (10%)	0.192
**Bioptic staging**				
- **Fibrosis F0**	11 (31.4%)	3 (11.1%)	3 (30%)	0.153
- **Fibrosis F1-3**	23 (65.7%)	22 (81.5%)	5 (50%)	0.146
- **Fibrosis F4**	1 (2.9%)	2 (7.4%)	2 (20%)	0.169
**Bioptic grading**				
- **Activity A1**	10 (29.4%)	5 (18.5%)	2 (20%)	0.583
- **Activity A2**	11 (32.4%)	5 (18.5%)	5 (50%)	0.156
- **Activity A3**	13 (38.2%)	17 (63.0%)	3 (30%)	0.083
**Histologic steatosis**				
- **No steatosis**	28 (80.0%)	23 (74.2%)	8 (80%)	0.858
- **Steatosis grade 1**	6 (17.1%)	4 (12.9%)	2 (20%)	0.927
- **Steatosis grade 2**	1 (2.9%)	0	0	0.585
**Treatment response group**				
- **Complete remission**	19 (48.7%)	12 (46.2%)	1 (11.1%)	0.113
- **Partial remission**	17 (43.6%)	11 (42.3%)	7 (77.8%)	0.147
- **Non-response**	3 (7.7%)	3 (11.5%)	1 (11.1%)	0.860
- **No therapy**	1	5	1	

Abbreviations: ALT, alanine aminotransferase; APRI, aspartate aminotransferase to platelet ratio index; BL, baseline; BMI, body-mass index; CRP, C-reactive protein; IgG, immunoglobulin G; LLN, lower limit of normal; ULN, upper limit of normal; and WBC, white blood cell count.

Supplementary Table S3. Characteristics of patients stratified according to TM6SF2-rs58542926 status, wildtype (E/E) vs. heterozygous (E/K) risk allele. Levels of significance: * p<0.05 (Mann–Whitney U test, Chi-square test).

	E/E (n=67)	E/K (n=14)	*p*-value
**Sex (m/f)**	19/48 (28.4% m)	4/10 (28.6% m)	0.453
**Age (years)**	56.3 (44.8-65.8)	60.2 (49.8-65.3)	0.648
**Cirrhosis at BL (n)**	10/67 (14.9%)	1/14 (7.1%)	0.439
**BMI (kg/m2)**	25.0 (21.6-28.0)	24.9 (21.7-26.2)	0.445
**Chol (mg/dl)**	191.3 (157.5-229.6)	162 (141-217.6)	0.214
**CRP (mg/dl)**	0.6 (0.1-1.1)	0.2 (0.0-1.0)	0.278
**Hb (g/dl)**	13.7 (12.9-14.7)	14.2 (12.4-15.7)	0.38
**Leu (G/L)**	5.5 (4.7-7.3)	6.3 (5.1-7.5)	0.277
**Thrombocytes (G/L)**	196 (158.5-234.5)	233.5 (197.5-271.3)	0.044*
**Albumin (x LLN)**	1.2 (1.1-1.3)	1.2 (1.0-1.3)	0.741
**PTZ (%)**	87 (70.4-99.1)	79.9 (69-92.5)	0.348
**ALT (x ULN)**	9.9 (3.2-20.1)	24.4 (1.1-33.0)	0.269
**Bili (x ULN)**	1.3 (0.7-4.0)	2.5 (0.9-7.0)	0.281
**CHE (x LLN)**	1.6 (0.9-2.0)	1.5 (1.1-1.9)	0.617
**APRI at 1y**	0.4 (0.3-0.7)	0.4 (0.2-0.5)	0.328
**IgG (mg/dl)**	2100 (1440-2810)	1720 (1410-1920)	0.132
**Diabetes mellitus (n)**	4/67 (6.0%)	1/14 (7.1%)	0.868
**Bioptic grading**			
- **Activity A1**	17 (29.3%)	3 (25.0%)	0.764
- **Activity A2**	16 (27.6%)	4 (33.3%)	0.688
- **Activity A3**	25 (43.1%)	5 (41.7%)	0.927
**Bioptic staging**			
- **Fibrosis grade 0**	16 (26.7%)	1 (8.3%)	0.172
- **Fibrosis grade 1-3**	39 (65.0%)	11 (91.7%)	0.067
- **Fibrosis grade 4**	5 (8.3%)	0	0.300
**Liver biopsy**			
- **No steatosis**	50 (83.3%)	9 (75.0%)	0.493
- **Steatosis grade 1**	10 (16.7%)	2 (16.7%)	1.0
- **Steatosis grade 2**	0	1 (8.3%)	0.024*
**Treatment response group**			
- **Complete remission**	26 (42.6%)	6 (46.2%)	0.816
- **Partial remission**	30 (49.2%)	5 (38.5%)	0.482
- **Non-response**	5 (8.2%)	2 (15.4%)	0.421
- **No therapy**	6	1	

Abbreviations: ALT, alanine aminotransferase; APRI, aspartate aminotransferase to platelet ratio index; BL, baseline; BMI, body-mass index; CRP, C-reactive protein; IgG, immunoglobulin G; LLN, lower limit of normal; ULN, upper limit of normal; and WBC, white blood cell count.

Supplementary Table S4. Characteristics of patients stratified according to MBOAT7-rs626238 status, wildtype (G/G) vs. heterozygous (G/C) vs. homozygous (C/C) risk allele. Levels of significance: * p<0.05 (Mann–Whitney U test, Chi-square test).

	G/G (n=25)	C/G (n=38)	C/C (n=18)	*p*-value
**Sex (m/f)**	3/22 (12.0% m)	14/24 (36.8% m)	6/12 (33.3% m)	0.088
**Age (years)**	55.0 (46.4-61.8)	56.0 (40.7-63.4)	60.3 (43.7-69.0)	0.527
**Cirrhosis at BL (n)**	3/25 (12.0%)	6/38 (15.8%)	2/18 (11.1%)	0.859
**BMI (kg/m2)**	24.5 (22.1-28.6)	25.2 (21.2-27.1)	25.1 (22.0-26.5)	0.767
**Cholesterol (mg/dl)**	195 (143-222.4)	191 (159-216.5)	183.5 (155.8-265)	0.995
**CRP (mg/dl)**	0.6 (0.0-1.1)	0.5 (0.1-1.0)	0.4 (0.0-1.0)	0.791
**Hemoglobin (g/dl)**	13.3 (11.6-14.2)	14.1 (13.7-15.0)	13.5 (12.7-14.5)	0.041*
**WBC (G/L)**	4.9 (4.0-6.6)	6.1 (5.1-8.1)	5.5 (4.9-6.4)	0.019*
**Thrombocytes (G/L)**	191 (163-250)	197 (159-242.5)	208 (184.5-233)	0.795
**Albumin (x LLN)**	1.2 (1.0-1.2)	1.2 (1.1-1.3)	1.2 (1.1-1.3)	0.464
**Prothrombin time (%)**	94.6 (75.5-103.8)	81.6 (69-94.3)	83.5 (68.5-92.9)	0.213
**ALT (x ULN)**	8.5 (5.9-20.1)	12.3 (3.9-28.1)	5.5 (1.5-19.4)	0.254
**Bilirubin (x ULN)**	1.0 (0.7-3.4)	1.3 (0.7-4.8)	1.5 (0.5-3.1)	0.832
**APRI after 1 year**	0.4 (0.3-0.6)	0.4 (0.3-0.6)	0.4 (0.3-0.7)	0.972
**IgG (mg/dl)**	1965 (1380-2800)	1840 (1510-2455)	22.6 (15.2-29.0)	0.726
**Diabetes mellitus (n)**	2/25 (8.0%)	3/38 (7.9%)	0	0.467
**Bioptic grading**				
- **Activity A1**	9 (42.9%)	4 (12.1%)	7 (43.8%)	0.016*
- **Activity A2**	2 (9.5%)	14 (42.4%)	4 (25.0%)	0.031*
- **Activity A3**	10 (47.6%)	15 (45.5%)	5 (31.3%)	0.558
**Bioptic staging**				
- **Fibrosis F0**	10 (45.5%)	3 (9.1%)	4 (23.5%)	0.008*
- **Fibrosis F1-3**	10 (45.5%)	28 (84.8%)	12 (70.6%)	0.008*
- **Fibrosis F4**	2 (9.1%)	2 (6.1%)	1 (5.9%)	0.893
**Liver biopsy**				
- **No steatosis**	19 (86.4%)	26 (78.8%)	14 (82.4%)	0.773
- **Steatosis grade 1**	3 (13.6%)	6 (18.2%)	3 (17.6%)	0.900
- **Steatosis grade 2**	0	1 (3.0%)	0	0.549
**Treatment response group**				
- **Complete remission**	11 (47.8%)	14 (38.9%)	7 (46.7%)	0.761
- **Partial remission**	12 (52.2%)	19 (52.8%)	4 (26.7%)	0.200
- **Non-response**	0	3 (8.3%)	4 (26.7%)	0.022*
- **No therapy**	2	2	3	0.362

Abbreviations: ALT, alanine aminotransferase; APRI, aspartate aminotransferase to platelet ratio index; BL, baseline; BMI, body-mass index; CRP, C-reactive protein; IgG, immunoglobulin G; LLN, lower limit of normal; ULN, upper limit of normal; and WBC, white blood cell count.

Supplementary Table S5. Characteristics of patients stratified according to HSD17B13-rs72613567:TA status, wildtype (A/A) vs. heterozygous (A/dupA) vs. homozygous (dupA/dupA) allele. Levels of significance: * p<0.05 (Mann–Whitney U test, Chi-square test).

	A/A (n=41)	A/dupA (n=29)	dupA/dupA (n=11)	*p*-value
**Sex (m/f)**	11/30 (26.8% m)	8/21 (27.6% m)	4/7 (36.4% m)	0.818
**Age (years)**	55.0 (44.8-65.8)	58.2 (46.4-62.4)	57.8 (51.8-68.7)	0.636
**Cirrhosis at BL (n)**	5/41 (12.2%)	5/29 (17.2%)	1/11 (9.1%)	0.746
**BMI (kg/m2)**	25.0 (21.8-28.6)	25.6 (22.6-26.9)	21.4 (20.3-26.7)	0.466
**Cholesterol (mg/dl)**	186 (142.9-255)	180.9 (158.3-208.5)	192.2 (163.1-215.8)	0.969
**CRP (mg/dl)**	0.6 (0.1-1.1)	0.3 (0.1-0.9)	0.4 (0.1-0.9)	0.788
**Hemoglobin (g/dl)**	13.7 (12.5-14.9)	14.1 (13.2-14.7)	13.6 (12.9-14.4)	0.794
**WBC (G/L)**	5.6 (4.9-7.2)	5.9 (4.7-7.8)	5.2 (4.4-6.5)	0.502
**Thrombocytes (G/L)**	194 (138-225.5)	225 (180-293)	200 (170.5-240.5)	0.067
**Albumin (x LLN)**	1.2 (1.0-1.3)	1.2 (1.2-1.3)	1.1 (1.0-1.2)	0.466
**Prothrombin time (%)**	87 (67-100)	85 (76.3-99.8)	80.9 (70.1-93.2)	0.719
**ALT (x ULN)**	9.9 (2.3-25.5)	9.6 (3.2-21.3)	15.8 (5.3-21.9)	0.840
**Bilirubin (x ULN)**	1.3 (0.8-5.0)	1.1 (0.6-3.4)	2.0 (0.8-5.0)	0.481
**APRI after 1 year**	0.4 (0.3-0.7)	0.4 (0.3-0.5)	0.4 (0.3-0.6)	0.908
**IgG (mg/dl)**	2060 (1450-2850)	1840 (1430-2280)	2020 (1500.3-2740)	0.872
**Diabetes mellitus (n)**	0	3/29 (10.3%)	2/11 (18.2%)	0.043*
**Bioptic grading**				
- **Activity A1**	11 (31.4%)	8 (29.6%)	1 (12.5%)	0.558
- **Activity A2**	9 (25.7%)	8 (29.6%)	3 (37.5%)	0.792
- **Activity A3**	15 (42.9%)	11 (40.7%)	4 (50.0%)	0.898
**Bioptic staging**				
- **Fibrosis grade 0**	8 (22.9%)	7 (25.0%)	2 (22.2%)	0.975
- **Fibrosis grade 1-3**	23 (65.7%)	20 (71.4%)	7 (77.8%)	0.750
- **Fibrosis grade 4**	4 (11.4%)	1 (3.6%)	0	0.324
**Liver biopsy**				
- **No steatosis**	29 (82.9%)	22 (78.6%)	8 (88.9%)	0.768
- **Steatosis grade 1**	6 (17.1%)	5 (17.9%)	1 (11.1%)	0.889
- **Steatosis grade 2**	0	1 (3.6%)	0	0.451
**Treatment response group**				
- **Complete remission**	15 (39.5%)	11 (44.0%)	6 (54.5%)	0.671
- **Partial remission**	18 (47.4%)	13 (52.0%)	4 (36.4%)	0.687
- **Non-response**	5 (13.2%)	1 (4.0%)	1 (9.1%)	0.477
- **No therapy**	3	4	0	

Abbreviations: ALT, alanine aminotransferase; APRI, aspartate aminotransferase to platelet ratio index; BL, baseline; BMI, body-mass index; CRP, C-reactive protein; IgG, immunoglobulin G; LLN, lower limit of normal; ULN, upper limit of normal; and WBC, white blood cell count.
